# Functional Characterization of Olfactory Proteins Involved in Chemoreception of *Galeruca daurica*

**DOI:** 10.3389/fphys.2021.678698

**Published:** 2021-06-09

**Authors:** Ling Li, Wen-Bing Zhang, Yan-Min Shan, Zhuo-Ran Zhang, Bao-Ping Pang

**Affiliations:** ^1^Research Center for Grassland Entomology, Inner Mongolia Agricultural University, Hohhot, China; ^2^Inner Mongolia Forestry and Grassland Pest Control and Quarantine Station, Hohhot, China

**Keywords:** *Galeruca daurica*, odorant-binding protein, chemosensory protein, fluorescence binding assay, RNA interference, electroantennogram

## Abstract

Odorant-binding proteins (OBPs) and chemosensory proteins (CSPs) play a fundamental role in insect olfaction. *Galeruca daurica* (Joannis) is a new pest with outbreak status in the Inner Mongolia grasslands, northern China. In this study, six olfactory protein genes (*GdauOBP1*, *GdauOBP6*, *GdauOBP10*, *GdauOBP15*, *GdauCSP4*, and *GdauCSP5*) were cloned by RACE and expressed by constructing a prokaryotic expression system. Their binding affinities to 13 compounds from host volatiles (*Allium mongolicum*) were determined by fluorescence-binding assay. In order to further explore the olfactory functions of *GdauOBP15* and *GdauCSP5*, RNA interference (RNAi) and electroantennogram (EAG) experiments were conducted. Ligand-binding assays showed that the binding properties of the six recombinant proteins to the tested volatiles were different. GdauOBP6, GdauOBP15, GdauCSP4, and GdauCSP5 could bind several tested ligands of host plants. It was suspected that GdauOBP6, GdauOBP15, GdauCSP4, and GdauCSP5 were related to the host location in *G. daurica.* We also found that there were different EAG responses between males and females when the *GdauOBP15* and *GdauCSP5* genes were silenced by RNAi. The EAG response of *G. daurica* females to 2-hexenal was significantly decreased in dsRNA-OBP15-injected treatment compared to the control, and the dsRNA-CSP5-treated females significantly reduced EAG response to eight tested host volatiles (1,3-dithiane, 2-hexenal, methyl benzoate, dimethyl trisulfide, myrcene, hexanal, 1,3,5-cycloheptatriene, and p-xylene). However, the EAG response had no significant difference in males. Both GdauOBP15 and GdauCSP5 may have different functions between males and females in *G. daurica* and may play more important roles in females searching for host plants.

## Introduction

Insects depend critically on olfactory systems to perceive various chemical signals in their environment (Forêt et al., 2007; Leal, 2013). As the primary sensory organs of insects, antennae are distributed with lots of sensilla hairs (Zacharuk, 1980). When the neurons in these sensilla are activated, they trigger a series of behavioral responses related to host recognition, oviposition, and mating (Martin et al., 2011; Hull et al., 2014). Odorant-binding proteins (OBPs) and chemosensory proteins (CSPs) have been identified in the lymph of chemosensilla (Vogt and Riddiford, 1981; McKenna et al., 1994). Both of them are small, hydrosoluble proteins that are expressed in the auxiliary cells of chemosensilla and secreted into the aqueous fluid around the olfactory neurons (Qiao et al., 2013). OBPs and CSPs are originally thought to be able to recognize and transport volatiles and lipophilic semiochemicals to the specific olfactory receptors (ORs) in the neuronal membrane across the aqueous sensillar lymph (Vogt et al., 2002; Leal, 2005; Pelosi et al., 2006). Many OBP and CSP genes have been identified so far; most of them were found in different tissues of insect, and some were even expressed in non-chemosensory organs (Gong et al., 2007; Dippel et al., 2014). They have also been implicated in embryonic development (Maleszka et al., 2007), larval ecdysis (Cheng et al., 2015), limb regeneration (Nomura et al., 1992), hematopoiesis, wound healing (Benoit et al., 2017), and humidity detection (Sun et al., 2018). Therefore, OBPs and CSPs conduct various tasks ranging from behavioral to multiple physiological and biological processes (Pelosi et al., 2017).

Fluorescence-binding assay is an efficient technique to study the binding properties of OBPs or CSPs to putative ligands and provide essential evidence for understanding their physiological function (Nathália et al., 2016). For example, fluorescence-binding assays showed that OasiCSP4, OasiCSP11, and OasiCSP12 of *Oedaleus asiaticus* showed a broad range of binding affinities to their host plant volatiles, fecal volatiles, and live body volatiles (Zhou et al., 2020). Wu et al. (2019) reported that CSP4 plays an important role during the process of transporting pheromones in *Apis mellifera* larva. The binding ability of AlinOBP11 in *Adelphocoris lineolatus* to non-volatile host plant secondary metabolites was preferential than that to volatile compounds, suggesting that AlinOBP11 could act as a carrier in the gustatory system (Sun et al., 2016). Recent studies have shown that the involvement of genes in olfactory functions can be ultimately impaired by silencing individual CSP or OBP genes to influence odor preference (Rebijith et al., 2016; Dong et al., 2017; Waris et al., 2018; Zhou et al., 2020). In addition, knowledge about the olfactory responses of insects to plant volatiles can provide strategies for pest management by identifying chemical signals (Das et al., 2013).

*Galeruca daurica* (Joannis) (Coleoptera: Chrysomelidae) is an oligophagous pest found in the Inner Mongolian grasslands of China in recent years (Yang et al., 2010; Hao et al., 2014). This pest has been reported to feed on the species of *Allium* plants, including *A. mongolicum*, *A. polyrhizum*, and *A. ramosum*, among which *A. mongolicum* is its favorite host (Hao et al., 2014, 2015). Extensive outbreaks of this pest have caused great losses to pasture in the Inner Mongolian grasslands since 2009, and the damage continues to increase (Zhou et al., 2019). This leaf beetle forages only *Allium* plants, implying an important role of olfaction in searching for specific host plants. However, little is known about the chemosensory mechanisms of this pest. Li et al. (2019) cloned *GdauOBP20* of *G. daurica* and clarified the binding property of the recombinant protein to main host plant volatiles. In this study, we selected *GdauOBP1*, *GdauOBP6*, *GdauOBP10*, *GdauOBP15*, *GdauCSP4*, and *GdauCSP5* (accession numbers: KX900453, KX900458, KX900462, KX900467, KY885474, and KY885475) for functional evaluation due to the fact that they were specifically highly expressed in antennae or in heads, and their expression levels were significantly different between males and females (Li et al., 2017, 2018). To clarify the function of these genes, the binding properties of four OBPs and two CSPs were analyzed using a number of ligands in competitive binding assays. Then, RNA interference (RNAi) was also used to reduce the expression levels of *GdauOBP15* and *GdauCSP5* in vivo, and the electroantennogram (EAG) response was recorded. Our present research aims to discover the molecular mechanisms of olfactory recognition and will provide a reference for pest management strategies.

## Materials and Methods

### Insects Rearing and Sample Collection

The larvae of *G. daurica* were collected from Xilinhot, Inner Mongolia, China (43°54′53′′N, 115°39′13′′E) in 2018, and reared at 26 ± 1°C, 60–80% relative humidity under a 16 h light: 8 h dark period.

### Cloning and Sequencing of Full-Length cDNA

Total RNA was extracted from the antennae of 3-day-old adults using the TaKaRa MiniBEST Universal RNA Extraction Kit (TaKaRa, Dalian, China). cDNA was synthesized using the PrimeScript^TM^ 1st Strand cDNA Synthesis Kit (TaKaRa, Dalian, China). Specific primers (Supplementary Table 1) were designed to the coding sequences of GdauOBP1, GdauOBP6, GdauOBP10, GdauOBP15, GdauCSP4, and GdauCSP5 in Primer Premier 5.0 based on the transcriptome database of *G. daurica* assembled in our laboratory. PCR amplifications were performed as 94°C for 3 min, followed by 30 cycles of 94°C for 30 s, 56°C for 30 s (each primer used a different annealing temperature), and 72°C for 1 min. Finally, it was extended for 10 min at 72°C. The amplified product was eluted (Gel DNA Mini Purification Kit, Tiangen, China) and cloned into the pMD19-T vector. Four positive transformants per individual were selected for plasmid isolation using MiniBEST Plasmid Purification Kit (TaKaRa, Dalian, China) and sent to the Beijing Liuhe Huada Gene Technology Company for sequencing in both forward and reverse directions.

The primers were designed for 5′ and 3′ RACE (Supplementary Table 2) based on obtained sequence fragments. 5′- and 3′-end amplifications were performed using SMARTer^®^ RACE 5′/3′ Kit (TaKaRa, Dalian, China) following the manufacturers’ protocol. Touchdown PCR (5 cycles of 94°C for 30 s, 72°C for 3 min followed by 5 cycles of 94°C for 30 s, 70°C for 30 s, 72°C for 3 min, and 25 cycles of 94°C for 30 s, 68°C for 30 s, 72°C for 2 min) and nested PCR (25 cycles of 94°C for 30 s, 68°C for 30 s, 72°C for 2 min) were conducted to enhance the amplification specificity of the 5′-UTR and 3′-UTR sequences. The amplified product was eluted and cloned as described in the previous section.

The sequence fragments and RACE sequence were assembled in DNAMAN 6.0 to obtain the full-length cDNA sequences. Open reading frames (ORFs) were obtained in ORF Finder^1^.

### Prokaryotic Expression and Purification of Recombinant Proteins

Gene-specific primers (containing restriction sites) (Supplementary Table 3) were designed to clone the coding regions of *GdauOBP1*, *GdauOBP6*, *GdauOBP10*, *GdauOBP15*, *GdauCSP4*, and *GdauCSP5*. The purified PCR products were cloned into the pMD19-T vector and transformed into *Escherichia coli* DH5α-competent cells. The validated plasmids were double-digested with the restriction enzymes and then ligated to the pET-28a (+) vector by T4 DNA ligase (New England BioLabs, NEB). The recombinant plasmids were transformed into *E. coli* BL21 Star (DE3)-competent cells. The positive clones were selected for mass culture at 37°C overnight. The recombinant proteins were induced with 1 mM isopropyl β-D-thiogalactoside (IPTG) and cultured for 4 h at 37°C.

After bacterial cells were centrifuged at 7,800 × *g* for 15 min, the precipitant was suspended in lysis buffer (50 mM NaH_2_PO_4_, 300 mM NaCl, 10 mM imidazole, pH 8.0). The cells were sonicated for 30 min on ice and centrifuged. The proteins were collected from the supernatant and precipitant, then detected by SDS-PAGE electrophoresis. The supernatant was filtered through a 0.45-μm filter and pumped into a Ni-NTA Agarose column (Qiagen, Germany). The eluted protein solution was dialyzed in a dialysis bag with a cutoff of 3,500 Da. The protein solutions were concentrated in ultrafiltration tubes with a cutoff of 10,000 Da. The concentration of the purified recombinant protein was measured using the BCA Assay Kit (Beyotime, Shanghai, China). The purified proteins were stored at −80°C until use.

### Fluorescence-Binding Assays

Fluorescence-binding assays were performed using a 970CRT spectrofluorophotometer (Hitachi, Japan). Instrument parameters were set as follows: Excitation slit of 10 nm, emission slit of 10 nm, sensitivity of 2 s, excitation wavelength of 337 nm, and scanning emission wavelength range of 350–700 nm. In order to measure the affinity of the fluorescent ligand 1-NPN (N-phenyl-1-naphthylamine) with the six recombinant proteins, the recombinant proteins were diluted to 2 μM in 50 mM Tris–HCl (pH 7.4) and added into a 1-cm light path quartz cuvette. Fluorescence values were recorded after the 1-NPN solution was added successively. The dissociation constant K_1–NPN_ was calculated using GraphPad Prism 7.0 software.

Thirteen main volatiles of the host plant (*A. mongolicum*) were selected as competing ligands for the fluorescence competitive binding assays (Table 1). All ligands were purchased from Sigma-Aldrich (St. Louis, MO, United States). These ligands were dissolved in methanol (HPLC grade) to make stock solutions of 1 mM, which were added into a 2-μM protein solution with 1-NPN saturated. The decrease in fluorescence intensity indicated that the bound 1-NPN was replaced by the ligands. Three duplications were used in the binding experiment. The curves were fitted using Scatchard plots. The dissociation constants (*Ki*) of the competitive ligands were calculated based on the equation: *K*_*i*_ = *IC50*/(1 + [1−*NPN*]/*K1*−*NPN*), where IC_50_ is the concentration of a competitor that results in a 50% reduction of the initial fluorescence intensity, and [1-NPN] and K_1–NPN_ are the free concentration of 1-NPN and the dissociation constant of the recombinant protein/1-NPN complex, respectively (Campanacci et al., 2001).

**TABLE 1:** List of odor samples.

Compound name	Molecular formula	Formula	CAS number	Purity (%)
Diallyl sulfide	C_6_H_10_S	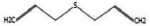	592-88-1	97
1,3-dithiane	C_4_H_8_S_2_	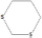	505-23-7	97
Dimethyl trisulfide	C_2_H_6_S_3_	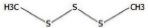	3658-80-8	98
Diallyl disulfide	C_6_H_10_S_2_	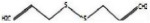	2179-57-9	98
Diallyl trisulfide	C_6_H_10_S_3_	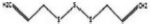	2050-87-5	98
Dimethyl disulfide	C_2_H_6_S_2_	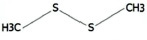	624-92-0	99
(Z)-2-Hexen-1-ol	C_6_H_12_O	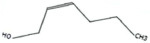	928-95-0	96
Myrcene	C_10_H_16_	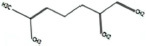	123-35-3	95
2-Hexenal	C_6_H_10_O	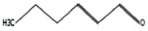	6728-26-3	97
Methyl benzoate	C_8_H_8_O_2_	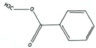	93-58-3	96
Hexanal	C_6_H_12_O	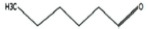	66-25-1	98
1,3,5-Cycloheptatriene	C_7_H_8_	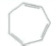	544-25-2	95
p-Xylene	C_8_H_10_	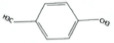	106-42-3	99

### RNA Interference of *GdauOBP15* and *GdauCSP5*

*GdauOBP15* and *GdauCSP5* were amplified by RT-PCR using specific primers containing the T7 promoter at the 5′ end (Supplementary Table 4). The purified PCR products of the two genes were subcloned into the pGEM-T vector and transformed into *E. coli* DH5α-competent cells. The plasmids verified by sequencing were used as templates to amplify the target sequence. The double-stranded RNAs (dsRNA) were synthesized using the T7 RiboMAX^TM^ Express RNAi System (Promega, United States) following the manufacturer’s instructions. The concentration of dsRNA was determined by NanoPhotometer^TM^ P-Class (Implen, Germany). The integrity was analyzed by 1.5% agarose gel electrophoresis. Finally, the dsRNA was diluted to 1,000 μg/μL in enzyme-free water and stored at −80°C. The double-stranded RNA of green fluorescent protein (GFP) was synthesized as control.

Two microliters of dsRNA (1,000 μg/μL) was injected into the intersegmental membrane between the fourth and fifth abdominal segments of 3-day-old adult females and males using a microinjector (Shimadzu, Japan). All the treated insects, including dsRNA-OBP15-injected, dsRNA-CSP5-injected, and dsRNA-GFP-injected, were reared under natural temperature conditions in the lab. Samples were taken 48 h later for interference efficiency measurement and electroantennogram analysis.

### Quantitative Real-Time PCR (qRT-PCR) Measurement

Antenna samples were collected after the treated insects were recovered for 48 h. Each treatment was performed with three biological replicates and 20 individuals per replicate. qRT-PCR was conducted using the FTC-3000P Real-Time Quantitative Thermal Cycler (Funglyn Biotech, Canada). BRYT^®^ Green dye (GoTaq^®^ qPCR Master Mix, Promega, United States) was used as the fluorescence reporter for each elongation cycle. qRT-PCR was conducted in a 10-μL reaction system with three technical replicates for each sample. All reactions were performed under the following conditions: denaturation at 95°C for 10 min, 45 cycles of 95°C for 15 s, and 60°C for 1 min, and finally a dissociation curve was analyzed. The succinate dehydrogenase complex (*SDHA*) gene of *G. daurica* was used as a reference gene (Tan et al., 2016). The relative expression levels of each gene were estimated by the 2^–ΔΔCT^ method (Livak and Schmittgen, 2001).

### Electroantennogram Analysis

Electroantennograms were conducted to record the antennal responses of dsRNA-OBP15-injected, dsRNA-CSP5-injected, and dsRNA-GFP-injected to 12 plant volatiles. These odorants were dissolved in dichloromethane (HPLC grade) to make stock solutions of 1 mol/L, and dichloromethane alone served as solvent control. The antennae were cut off at the base, and the ends were removed then attached to the electrode with electrode gel. Filter paper strips (1 cm × 0.5 cm) were loaded with 10 μL of each chemical solution and then inserted into a Pasteur pipette. The tube was connected to an air stimulus controller (CS-55; SynTech). The signals were detected by a high-impedance amplifier (IDAC-2; SynTech) and analyzed using SynTech software (GC-EAD 2014 v1.2.5). The pulse duration time was 0.2 s with a stimulation interval of 30 s, and each compound was tested three times for each antenna. It was taken as the absolute value of the difference value between the maximum amplitude achieved by the odor stimulus and the baseline level. Each antenna was stimulated three times. All EAG results are presented as the mean EAG values from six female or male antennae.

### Statistical Analysis

The binding assay curve was fitted by GraphPad Prism 7.0, using the least squares (ordinary) fit of the second-order polynomial. Data from qPCR and EAG assays were analyzed using SPSS Statistics 17.0. *T*-test (*P* < 0.05) was used to analyze the differences among the groups.

## Results

### Gene Cloning

**FIGURE 1:**
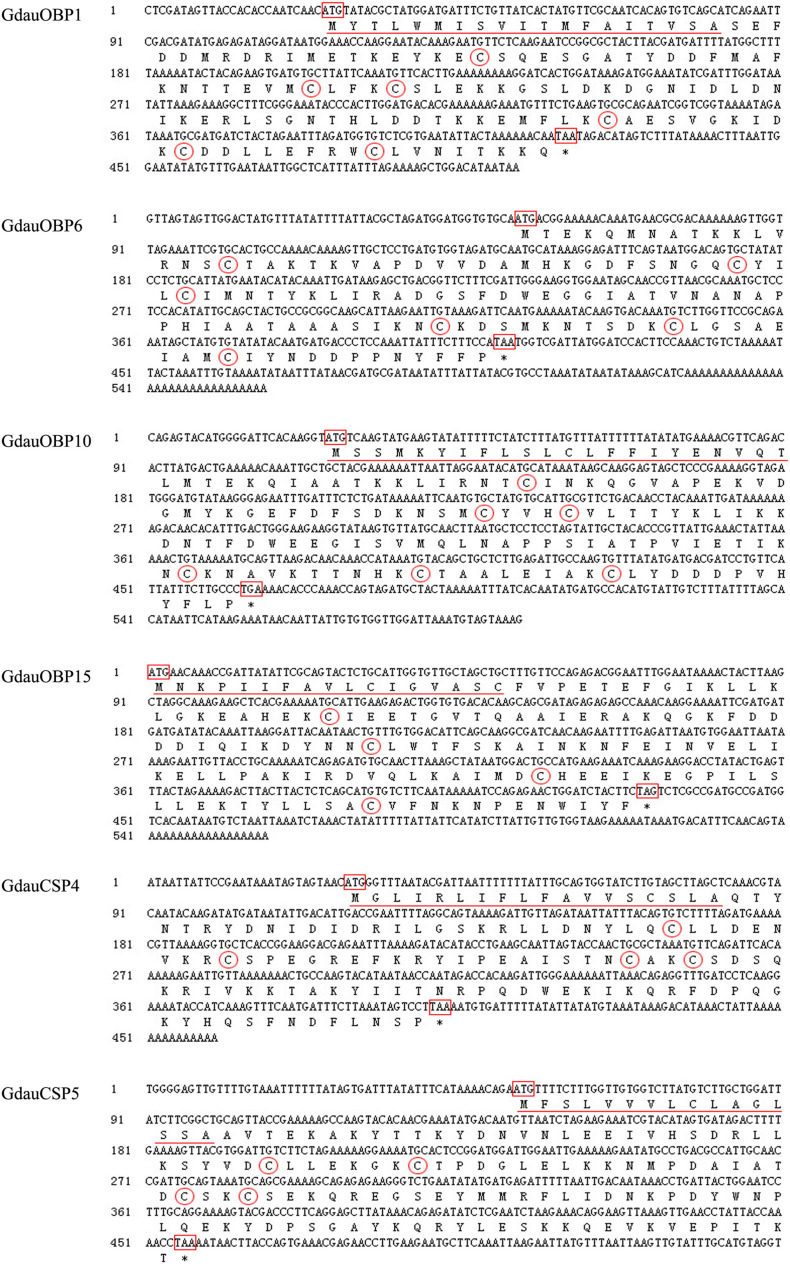
Nucleotide and amino acid sequences of *GdauOBP1*, *GdauOBP6*, *GdauOBP10*, *GdauOBP15*, *GdauCSP4*, and *GdauCSP5.* The start and stop codons are boxed, signal peptide is underlined, conversed cysteine residues are circled, and asterisks indicate the termination of translation.

Four OBP genes (*GdauOBP1*, *GdauOBP6*, *GdauOBP10*, and *GdauOBP15*) and two CSP genes (*GdauCSP4* and *GdauCSP5*) were cloned from *G. daurica* using RT-PCR and RACE-PCR strategies. Nucleotide and amino acid sequences are shown in Figure 1. The full-length *GdauOBP1* complementary DNA (cDNA) consisted of 503 bp with an ORF of 396 bp. The lengths of *GdauOBP6* and *GdauOBP10* were 557 bp with an ORF of 360 and 593 bp with an ORF of 441 bp, respectively. The 5′-end cDNA cloning of *GdauOBP15* failed, and the ORF was 360 bp in length. The lengths of *GdauCSP4* and *GdauCSP4* were 460 bp with an ORF of 375 and 540 bp with an ORF of 405 bp, respectively.

### Protein Expression and Purification

**FIGURE 2:**
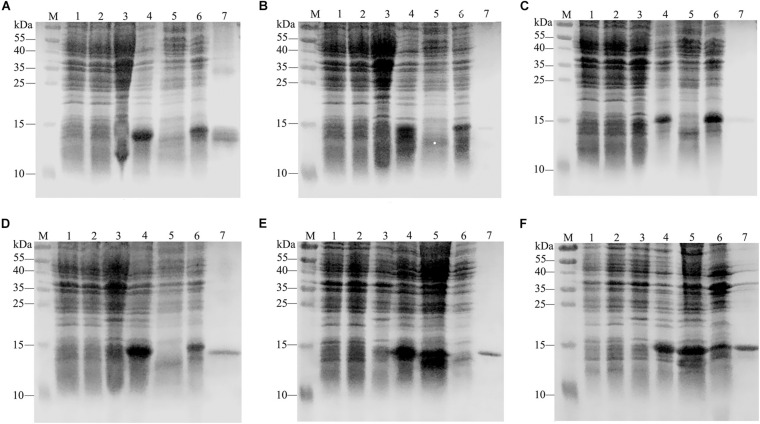
SDS-PAGE analyses showing the expression and purification of four recombinant GdauOBPs and two GdauCSP proteins. **(A)** GdauOBP1; **(B)** GdauOBP6; **(C)** GdauOBP10; **(D)** GdauOBP15; **(E)** GdauCSP4; **(F)** GdauCSP5; M, Protein molecular weight marker; 1, non-induced pET-28a (+); 2, induced pET-28a (+); 3, non-induced recombinant proteins; 4, induced recombinant proteins; 5, supernatant after sonication; 6, inclusion body after sonication; 7, purified recombinant protein.

The calculated molecular weights of GdauOBP1, GdauOBP6, GdauOBP10, GdauOBP15, GdauCSP4, and GdauCSP5 were 15.19, 12.93, 16.64, 16.33, 14.55, and 15.32 kDa, respectively (Li et al., 2017, 2018). The recombinant proteins were successfully expressed in the *E. coli* expression system. The SDS-PAGE analysis indicated that GdauCSP4 and GdauCSP5 were mainly detected in the medium supernatant, while GdauOBP1, GdauOBP6, GdauOBP10, and GdauOBP15 were mainly detected in the precipitate (Figure 2). Since all four GdauOBPs were mainly expressed in inclusion bodies, urea was added to solubilize and denature the recombinant proteins, followed by extensive dialysis to renature them, as described previously (Calvello et al., 2003; Zhang et al., 2012). The concentrations of the purified proteins were 1.02, 0.14, 0.19, 0.43, 0.58, and 0.74 mg/mL for GdauOBP1, GdauOBP6, GdauOBP10, GdauOBP15, GdauCSP4, and GdauCSP5, respectively.

### Ligand-Binding Assays

**FIGURE 3:**
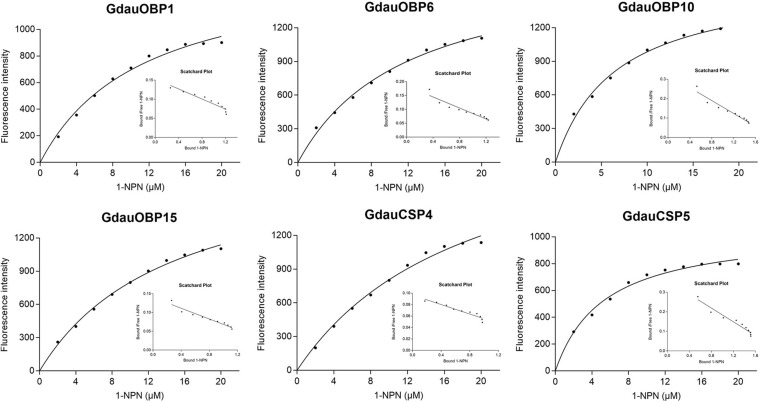
Binding curves and Scatchard plot analysis of 1-NPN to GdauOBP1, GdauOBP6, GdauOBP10, GdauOBP15, GdauCSP4, and GdauCSP5 at pH 7.4.

**FIGURE 4:**
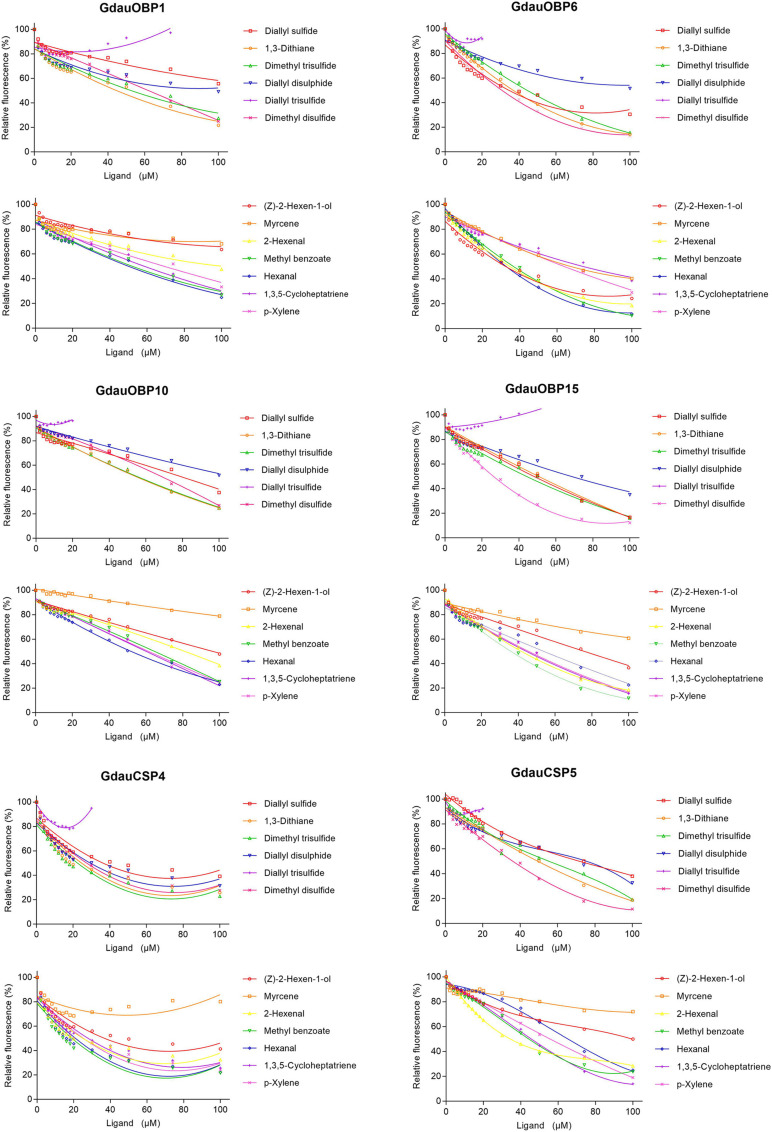
Competitive binding curves of GdauOBP1, GdauOBP6, GdauOBP10, GdauOBP15, GdauCSP4, and GdauCSP5 with thirteen ligands from the host plant volatiles.

The dissociation constants of six recombinant proteins with 1-NPN were measured, with GdauOBP1, GdauOBP6, GdauOBP10, GdauOBP15, GdauCSP4, and GdauCSP5 of 11.36, 11.83, 6.54, 14.59, 18.87, and 5.34 μM, respectively, suggesting that 1-NPN is an appropriate reporter to these proteins. The binding curves and Scatchard plots are shown in Figure 3. 1-NPN was used as the fluorescent reporter to measure the affinity of six recombinant proteins with 13 host plant volatiles in competitive binding assays. The results are shown in Table 2 and Figure 4. GdauOBP1 and GdauOBP10 had weak or no binding affinity to all tested compounds (*Ki* > 30 μM). GdauOBP6 showed strong binding affinity to dimethyl disulfide, hexanal, 2-hexenal, and (Z)-2-hexen-1-ol, with *Ki* values of 25.65, 27.72, 28.37, and 29.29 μM, respectively. GdauOBP15 bound to dimethyl disulfide specifically with the *Ki* value of 23.09 μM. The binding affinities of the two CSPs to the host plant volatiles varied greatly. GdauCSP4 showed a broad binding profile with nine compounds (methyl benzoate, hexanal, dimethyl trisulfide, 2-hexenal, 1,3-dithiane, p-xylene, dimethyl disulfide, 1,3,5-cycloheptatriene, and diallyl disulfide) with *Ki* values between 12.06 and 21.32 μM, among which the ligand with the strongest binding ability was methyl benzoate, followed by hexanal. However, GdauCSP5 could only bind dimethyl disulfide and 2-hexenal with the *Ki* values of 26.47 μM and 25.28 μM, respectively.

**TABLE 2:** Fluorescence competitive binding affinities of four recombinant GdauOBPs and two GdauCSP proteins with different ligands.

	GdauOBP1		GdauOBP6		GdauOBP10		GdauOBP15		GdauCSP4		GdauCSP5	
Ligand name	IC_50_	*Ki*	IC_50_	*Ki*	IC_50_	*Ki*	IC_50_	*Ki*	IC_50_	*Ki*	IC_50_	*Ki*
Diallyl sulfide	–	–	55.03	47.29	77.82	60.13	48.07	42.41	43.79	39.69	72.23	53.09
1,3-dithiane	70.65	60.34	36.66	31.50	59.45	45.93	57.18	50.46	18.46	16.73	49.99	36.74
Dimethyl trisulfide	63.20	53.98	44.45	38.19	67.40	52.07	56.69	50.03	16.58	15.03	53.71	39.47
Diallyl disulfide	97.08	82.91	72.13	61.98	–	–	42.30	37.32	23.52	21.32	61.57	45.25
Diallyl trisulfide	–	–	–	–	–	–	–	–	–	–	–	–
Dimethyl disulfide	64.87	55.41	29.85	25.65	61.87	47.80	26.17	23.09	23.12	20.96	36.02	26.47
(Z)-2-hexen-1-ol	86.39	73.78	33.02	28.37	95.58	73.84	76.04	67.10	49.96	45.29	–	73.49
Myrcene	82.62	70.56	68.71	59.04	–	–	–	–	–	–	84.17	61.86
2-hexenal	95.89	81.89	33.86	29.09	85.47	66.03	41.74	36.83	17.55	15.91	34.40	25.28
Methyl benzoate	65.37	55.83	36.44	31.31	73.92	57.11	35.87	31.65	13.30	12.06	44.30	32.56
Hexanal	70.78	60.45	32.26	27.72	52.01	40.18	68.77	60.69	15.41	13.97	61.44	45.15
1,3,5-cycloheptatriene	63.02	53.82	80.51	69.17	59.85	46.24	51.66	45.59	23.41	21.22	45.51	33.45
p-xylene	84.67	72.32	72.51	62.30	58.03	44.83	49.90	44.03	19.74	17.90	55.71	40.94

*“–” no binding.*

### Efficiency Analysis of RNAi on Expression Levels of *GdauOBP15* and *GdauCSP5*

**FIGURE 5:**
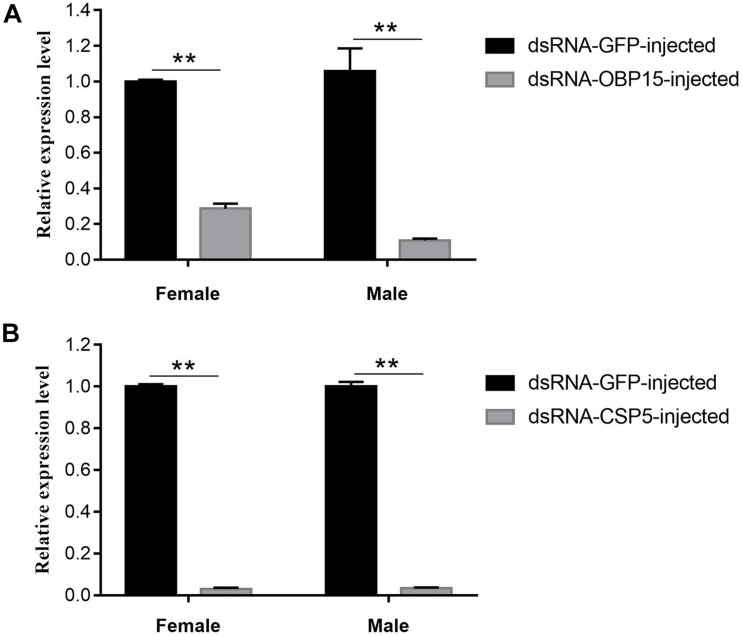
Relative expression levels of *GdauOBP15* **(A)** and *GdauCSP5* **(B)** at 48 h after dsRNA injection in *G. daurica* antennae measured by qRT-PCR (*t*-test; **P < 0.01). Columns indicate the mean ± standard error of three independent experiments.

The *GdauOBP15* and *GdauCSP5* genes were silenced by RNAi to elucidate their biological functions in vivo. The efficiency of gene silencing at 48 h with 2,000 μg/μL was investigated by qRT-PCR. The results revealed that the injection of dsRNA-OBP15 and dsRNA-CSP5 significantly reduced the expression levels of *OBP15* and *CSP5* in both male and female antenna, compared with non-target control groups (*P* < 0.01) within 48 h in *G. daurica* (Figure 5). RNAi reduced the expression levels of *GdauOBP15* to 28.65% and 10.74% in females and males, and the expression levels of *GdauCSP5* were reduced to 2.93 and 3.31% in females and males, respectively.

### Effect of RNAi on Electroantennogram Recording to *G. daurica*

**FIGURE 6:**
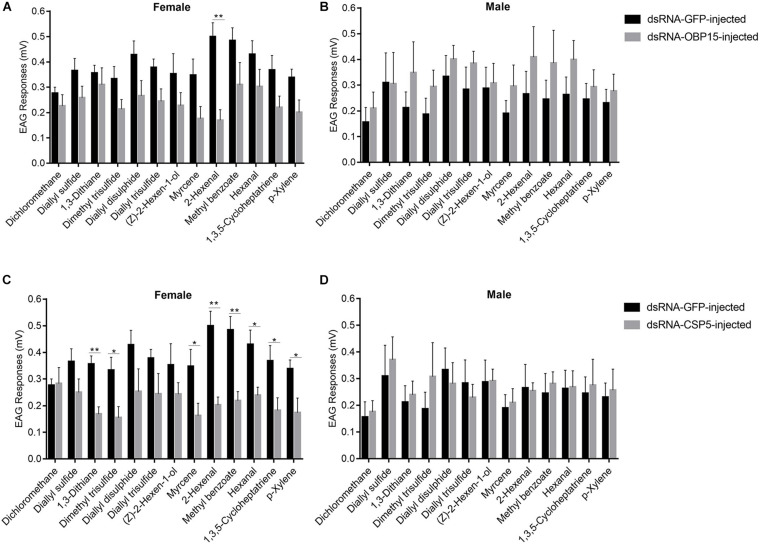
EAG responses of dsRNA-GdauOBP15-injected **(A,B)** and dsRNA-GdauCSP5-injected **(C,D)** in *G. daurica* female and male to various compounds (*t*-test; *P < 0.05; **P < 0.01). Columns indicate the mean ± standard error of six independent experiments.

We examined the responses of non-target control and dsRNA-treated (*GdauOBP15* and *GdauCSP5*) *G. daurica* adults to host plant volatiles. It was found that there were different EAG responses between males and females when the *GdauOBP15* and *GdauCSP5* genes were silenced by RNAi. The electrophysiological responses of *G. daurica* to all tested volatiles were decreased in dsRNA-OBP15-injected females compared to the control, and the response to 2-hexenal was reduced significantly (*P* < 0.01) among them (Figure 6A), whereas it increased in the injected males but not significantly (*P* > 0.05) (Figure 6B). Antennae of dsRNA-CSP5-injected females showed significantly lower electrophysiological responses to eight volatiles, 1,3-dithiane, 2-hexenal, methyl benzoate (*P* < 0.01), dimethyl trisulfide, myrcene, hexanal, 1,3,5-cycloheptatriene, and p-xylene (0.01 < *P* < 0.05) (Figure 6C). The EAG values of dsRNA-CSP5-injected males to most volatiles were increased but not significantly (*P* > 0.05) (Figure 6D).

## Discussion

In this study, four OBP genes (*GdauOBP1*, *GdauOBP6*, *GdauOBP10*, and *GdauOBP15*) and two CSP genes (*GdauCSP4* and *GdauCSP5*) were cloned from *G. daurica* using RT-PCR and RACE-PCR strategies. The results of ORF sequences were consistent with those identified in the transcriptome (Li et al., 2017, 2018). This experiment has laid a reliable foundation for subsequent experiments.

*G. daurica* feeds only on *Allium* plants, implying an important role of olfaction in searching for specific host plants. *A. mongolicum* is its favorite food (Hao et al., 2014, 2015). Therefore, 13 main representative components were selected from the *A. mongolicum* volatiles for this study. Fluorescence-binding assays showed that GdauOBP1 and GdauOBP10 had weak or no binding affinity to all tested compounds. It is necessary to expand the testing range of ligands in order to obtain the ligands with high affinity, such as sex pheromones and aggregation pheromones. For instance, GmolGOBP2 could not effectively bind host plant volatiles but showed a specific binding affinity for dodecanol, a minor sex pheromone component of *Grapholita molesta* (Li et al., 2016). PxylGOBP1 and PxylGOBP2 showed binding affinities to the sex pheromone of *Plutella xylostella* (Cai et al., 2020). There was another possibility that the structures and functions of the proteins might be affected by the expression and purification methods. GdauOBP6, GdauOBP15, GdauCSP4, and GdauCSP5 could bind several tested ligands of host plants. Among them, dimethyl disulfide, diallyl disulfide, 1,3-dithiane, and dimethyl trisulfide are sulfocompounds with a strong pungent odor, which are the symbolic components of *Allium* plants in Liliaceae such as onion and garlic (He et al., 2004; Wuren, 2011; Cheng et al., 2014; Li et al., 2015). The olfactory neurons of the basiconica sensilla on locust mouthpart could be directly activated by 2-hexenal and hexanal. In addition, hexanal could also enhance the transmission of nerve signals and regulate the ability of locusts to recognize odors (Zhang et al., 2017). Combined with our previous studies that these four genes were highly expressed in antenna (Li et al., 2017, 2018), the proteins coded by these genes could selectively bind to host volatiles, suggesting that they may be involved in host plant localization. For example, the *MaltCSP5* gene of *Monochamus alternatus* was mainly expressed in male and female antenna, and the binding affinity of recombinant CSP5 showed very strong binding abilities to pine plant volatiles (Ali et al., 2019). Zeng et al. (2018) found that CmedCSP1 and CmedCSP2 were localized in basiconica sensilla and showed strong binding affinities with a wide range of host-related semiochemicals. Injecting target dsRNAs resulted in a significant decrease in EAG responses evoked by the host volatiles of *Cnaphalocrocis medinalis*.

The importance of GdauOBP15 and GdauCSP5 in binding to major volatiles was further confirmed by our RNAi experiment. The injection of dsRNA-OBP15 and dsRNA-CSP5 significantly decreased the *GdauOBP15* and *GdauCSP5* expression levels. The electrophysiological response of *G. daurica* females to 2-hexenal was significantly decreased in dsRNA-OBP15-injected treatment compared to the control, and antennae of dsRNA-CSP5-treated females significantly reduced EAG response to eight tested host volatiles (1,3-dithiane, 2-hexenal, methyl benzoate, dimethyl trisulfide, myrcene, hexanal, 1,3,5-cycloheptatriene, and p-xylene). Our results corresponded with previous studies that silencing CSP or OBP genes can affect odor preferences (Rebijith et al., 2016; Dong et al., 2017; Waris et al., 2018; Zhou et al., 2019). Zhang et al. (2016) reported that silencing *AlinOBP4* by RNAi induced declining electrophysiological responses of *Adelphocoris lineolatus* to sex pheromone components and some host plant volatiles. Further study showed that the binding property of the recombinant AlinOBP4 protein was consistent with the results of RNAi (Wang et al., 2020). However, our results of EAG response after RNAi were inconsistent with the fluorescence-binding assays. GdauOBP15 had weak binding affinity to 2-hexenal, and GdauCSP5 could only bind dimethyl disulfide and 2-hexenal. The reason might be that they cooperate with multiple binding proteins to transport numerous compounds. For example, in *Anopheles gambiae*, OBP1 and OBP4 were co-expressed and formed heterodimers in the sensillum lymph, which showed different binding properties from the individual proteins (Qiao et al., 2011). In *A. lineolatus*, a mixture of AlinCSP5 and AlinCSP6 enhanced the binding affinity to terpenoids which did not bind with individuals (Sun et al., 2015). There was no significant change in the males’ electrophysiological responses to host plant volatiles after RNAi. Our previous study showed that there were significant differences in the expression levels of *GdauOBP15* and *GdauCSP5* in male and female antennae (Li et al., 2018). This implies that GdauOBP15 and GdauCSP5 may have different functions in odor perception between males and females in *G. daurica*, and they play more important roles in females searching for host plants, but this needs further study for confirmation.

In this study, we tried to combine molecular and electrophysiological methods to clarify the functions of several GdauOBPs and GdauCSPs in *G. daurica*. We hypothesize that GdauOBP6, GdauOBP15, GdauCSP4, and GdauCSP5 may be involved in host recognition in the adult chemosensory system. The reduction in *GdauOBP15* and *GdauCSP5* transcript abundance leads to a decrease in the female electrophysiological responses to host plant volatiles. These discoveries provided important clues for revealing the molecular mechanism of host selection of *G. daurica* and will facilitate the development of effective volatile attractants, which provide the possibility for pest monitoring and biological control.

## Data Availability Statement

The raw data supporting the conclusions of this article will be made available by the authors, without undue reservation.

## Ethics Statement

The animal subject used in this study is a leaf beetle in grasslands, which is an invertebrate and exempt from this requirement. No specific permits were required for the beetle’s collection from the field and for maintenance in laboratory. This study did not involve any endangered species, protected species, or protected areas.

## Author Contributions

LL and B-PP conceived and designed the experiments. LL wrote the manuscript. LL, W-BZ, Y-MS, and Z-RZ performed the experiments. B-PP provided valuable suggestions and helped to revise the manuscript. All authors discussed the results and approved the final manuscript.

## Conflict of Interest

The authors declare that the research was conducted in the absence of any commercial or financial relationships that could be construed as a potential conflict of interest.

## Footnotes

1
https://www.ncbi.nlm.nih.gov/orffinder/
