# Correction: Functional characterization of olfactory proteins involved in chemoreception of *Galeruca daurica*

**DOI:** 10.3389/fphys.2025.1654902

**Published:** 2025-07-23

**Authors:** Ling Li, Wen-Bing Zhang, Yan-Min Shan, Zhuo-Ran Zhang, Bao-Ping Pang

**Affiliations:** ^1^ Research Center for Grassland Entomology, Inner Mongolia Agricultural University, Hohhot, China; ^2^ Inner Mongolia Forestry and Grassland Pest Control and Quarantine Station, Hohhot, China

**Keywords:** *Galeruca daurica*, odorant-binding protein, chemosensory protein, fluorescence binding assay, RNA interference, electroantennogram

Incorrect supplementary material:

“File **Supplementary Table S4** was erroneously published with the original version of this paper.”

The file has now been replaced.

The original article has been updated.

## Supplementary material

The Supplemnentary Material for this article can be found online at: https://www.frontiersin.org/articles/10.3389/fphys.2025.1654902/full#supplementary-material

