# Supplementary material for: Functional Characterization of Olfactory Proteins Involved in Chemoreception of Galeruca daurica
Source: Front Physiol. 2021 Jun 9;12:678698. doi: 10.3389/fphys.2021.678698 (PMC8221581; doi:10.3389/fphys.2021.678698)
Supplement: Supplementary file 1 [file Table_1.DOCX]

Table. S1 List of gene cloning primer

| Gene names | Forward primer (5' to 3') | Reverse primer (5' to 3') |
| --- | --- | --- |
| *OBP1* | ATGTATACGCTATGGAT | TTATTGTTTTTTAGTAATATTCA |
| *OBP6* | ATGACGGAAAAACAAATGA | TTATGGAAAGAAATAATTTG |
| *OBP10* | Atgtcaagtatgaagtatatt | TCAGGGCAAGAAATAATG |
| *OBP15* | ATGAACAAACCGATTATATT | CTAGAAGTAGATCCAGT |
| *CSP4* | ATGGGTTTAATACGATTAAT | TTAAGGACTATTTAAGAAATC |
| *CSP5* | ATGTTTTCTTTGGTTGTGG | TTAGGTTTTGGTAATAGGTT |
