# Supplementary material for: Functional Characterization of Olfactory Proteins Involved in Chemoreception of Galeruca daurica
Source: Front Physiol. 2021 Jun 9;12:678698. doi: 10.3389/fphys.2021.678698 (PMC8221581; doi:10.3389/fphys.2021.678698)
Supplement: Supplementary file 2 [file Table_2.DOCX]

Table.S2 List of RACE cloning primer

| Primer names | 5'-RACE primer (5' to 3') | 3'-RACE primer (5' to 3') |
| --- | --- | --- |
| *OBP1* GSP | TCATATCGTCGAATTCTGATGCTGA | AGAATTTAGATGGTGTCTCGTGAAT |
| *OBP1* NGSP | CATCGTAAGTAGCGCCGGATTCTTG | TTCTGAAGTGCGCAGAATCGGTCGG |
| *OBP6* GSP | TCAGGAGCAACTTTTGTTTTGGCAG | TGACAAATGTCTTGGTTCCGCAGAA |
| *OBP6* NGSP | TCCCAATCGAAAGAACCGTCAGCTC | CTGCCGCGGCAAGCATTAAGAATTG |
| *OBP10* GSP | TTTCGTAGCAGCAATTTGTTTTTCA | GTACAGCTGCTCTTGAGATTGCCAA |
| *OBP10* NGSP | CATCCCATCTACCTTTTCGGGAGCT | GCTCCTCCTAGTATTGCTACACCCG |
| *OBP15* GSP | TTTTCGTGAGCTTCTTTGCCTAGCT | TGCAAAAATCAGAGATGTGCAACTT |
| *OBP15* NGSP | TCGCTGCTTGTGTCACACCAGTCTC | TGGACATTCAGCAAGGCGATCAACA |
| *CSP4* GSP | CTGCCTAAAATTCGGTCAATGTCAA | CCAATAGACCACAAGATTGGGAAAA |
| *CSP4* NGSP | TCCTTCCGGTGAGCACCTTTTAACG | CCTGAAGCAATTAGTACCAACTGCG |
| *CSP5* GSP | TTCGTTGTGTACTTGGCTTTTTCGG | ACGACCCTTCAGGAGCTTATAAACA |
| *CSP*5 NGSP | CCAATCCATCCGGAGTGCATTTTCC | TGCAGCGAAAAGCAGAGAGAAGGGT |
