# Supplementary material for: Functional Characterization of Olfactory Proteins Involved in Chemoreception of Galeruca daurica
Source: Front Physiol. 2021 Jun 9;12:678698. doi: 10.3389/fphys.2021.678698 (PMC8221581; doi:10.3389/fphys.2021.678698)
Supplement: Supplementary file 3 [file Table_3.DOCX]

Table S3 The primers for prokaryotic expression

| Gene names | Forward primer (5' to 3') | Reverse primer (5' to 3') | Restriction enzyme |
| --- | --- | --- | --- |
| *OBP1* | GGATCCTCAGAATTCGACGAT | CTCGAGTTATTGTTTTTTAGTAATATTCA | *Bam*H I *Xho* I |
| *OBP6* | GAATTCATGACGGAAAAACAAATGA | CTCGAGTTATGGAAAGAAATAATTTG | *Eco*R I *Xho* I |
| *OBP10* | GGATCCCTTATGACTGAAAAACAA | CTCGAGTCAGGGCAAGAAATA | *Bam*H I *Xho* I |
| *OBP15* | GAATTCTTTGTTCCAGAGACGG | CTCGAGCTAGAAGTAGATCCAGT | *Eco*R I *Xho* I |
| *CSP4* | GAATTCCAAACGTACAATACAAGATATGATA | CTCGAGTTAAGGACTATTTAAGAA | *Eco*R I *Xho* I |
| *CSP5* | GAATTCGCAGTTACCGAAAAAGCCAAGTA | CTCGAGTTAGGTTTTGGTAATAGG | *Eco*R I *Xho* I |

The restriction sites are underlined
