# Supplementary material for: Functional Characterization of Olfactory Proteins Involved in Chemoreception of Galeruca daurica
Source: Front Physiol. 2021 Jun 9;12:678698. doi: 10.3389/fphys.2021.678698 (PMC8221581; doi:10.3389/fphys.2021.678698)
Supplement: Supplementary file 4 [file Table_4.docx]

Table S4 List of RNAi primers

| Genes | Forward primer (5' to 3') | Reverse primer (5' to 3') |
| --- | --- | --- |
| GdauOBP15 | TAATACGACTCACTATAGGTTTGTTCCAGAGACGGAAT | TAATACGACTCACTATAGGCTAGAAGTAGATCCAGTTCT |
| GdauCSP5 | TAATACGACTCACTATAGGGCAGTTACCGAAAAAGCCAAGTA | TAATACGACTCACTATAGGTTAGGTTTTGGTAATAGGTTCAACT |
| GFP | TAATACGACTCACTATAGGGCACAAGTTCAGCGTGTCCG | TAATACGACTCACTATAGGGTTCACCTTGATGCCGTTC |
